# Clients’ Experiences With Internet-Based Psychological Treatments for Mental Disorders: Protocol for a Metasynthesis of Qualitative Studies

**DOI:** 10.2196/resprot.9722

**Published:** 2018-11-21

**Authors:** Javier Fernández-Álvarez, Amanda Díaz-García, Mª Dolores Vara, Guadalupe Molinari, Desirée Colombo, Giuseppe Riva, Rosa M Baños, Cristina Botella

**Affiliations:** 1 Università Cattolica del Sacro Cuore Milan Italy; 2 Universitat Jaume I Castellon Spain; 3 Universitat de Valencia Valencia Spain; 4 Applied Technology for Neuro-Psychology Laboratory, Instituto Auxologico Italiano Milan Italy

**Keywords:** barriers, clients’ experiences, facilitators, internet-based treatment, metasynthesis, qualitative

## Abstract

**Background:**

Given the rise of internet-based treatments as an effective therapeutic tool for psychological disorders, it is necessary to carry out research that examines clients’ experiences with this type of intervention. The qualitative methodology has been found to be useful for analyzing clients’ perceptions in terms of facilitators and barriers, acceptability, and negative effects of internet-based treatments. However, a lack of integration of these primary studies has prevented their findings from being applied to new research and in clinical practice.

**Objective:**

The objective of this paper is to describe the protocol for a metasynthesis of qualitative studies exploring the experiences of clients who underwent an internet-based treatment.

**Methods:**

Elliot and Timulak’s metasynthesis approach will be used to review and synthesize qualitative studies related to client experiences in terms of the barriers and facilitators they perceived when undergoing internet-based treatment. For each search string, the features in the Sample, Phenomenon of Interest, Design, Evaluation, Research type (SPIDER) tool will be considered. Electronic databases (PubMed, PsycINFO, and Web of Science) will be searched. Two independent reviewers will analyze the material in order to determine whether the eligibility criteria are fulfilled. Findings will make it possible to create a hierarchy of domains in terms of their relevance across all the primary studies. The data obtained from primary studies will be cross-analyzed using descriptive and interpretative procedures.

**Results:**

The search strategy is currently being conducted. First results are expected to be submitted for publication in 2019.

**Conclusions:**

We will develop conceptual framework of the barriers and facilitators perceived by clients and propose their implications and recommendations for clinical practice, research, and training.

**Trial Registration:**

PROSPERO CRD42018079894; https://www.crd.york.ac.uk/prospero/display_record.php?RecordID=79894 (Archived by WebCite at http://www.webcitation.org/73C6OtlS7).

**International Registered Report Identifier (IRRID):**

PRR1-10.2196/9722

## Introduction

### Background

The high prevalence rates of psychological disorders are one of the main public health concerns in contemporary society [1,2]. Although well-established, effective psychological methods exist for the treatment of numerous disorders [3], a vast array of problems still persist, including relapse rates [4], dropout rates [5], iatrogenic and inert treatments [6], and the well-established gap between science and practice [7,8].

Specifically, the dissemination of evidence-based psychological treatments has been the subject of heated debate over the past decade [9]**.** The lack of economic resources, remote geographical settings, or the enduring the social stigma of psychological disorders is among the barriers preventing access to mental health treatment [10]**.**

In this scenario, internet-based treatments (IBTs) have emerged as a useful alternative to cope with the challenge of disseminating psychological treatments [11]. In recent years, these types of treatments have shown ample evidence of their efficacy and effectiveness for a wide range of psychological disorders and medical conditions. The most important aspect of IBTs is their potential to improve cost-effectiveness compared with face-to-face traditional therapy [12].

Early IBTs were computer-based but not delivered over the internet [13,14]. Current interventions are available over the internet and often recognized as internet-based therapies. Among the vast array of internet-based therapies or IBTs available, important differences among them must be mentioned: Some of these therapies are self-applied treatment protocols, which serve to reduce therapist support as much as possible and are sometimes even unguided. Other IBTs include some degree of therapist support, which can be delivered through emails, short message service text messages, phones calls, or videoconferences. Also, there are blended treatments, which are a combination of a self-applied IBTs and regular contact with a therapist at different points in time during the therapeutic process. Some studies also consider videoconferencing to be another kind of internet-based therapy that can be delivered through different devices, mainly computers [15,16]. Finally, smartphone-based interventions are becoming useful tools for improving access, and potentially the effectiveness, of treatments [17,18].

Apart from the numerous studies focusing on the extent to which IBTs are useful for providing psychological treatments, a considerable amount of research has also examined client experiences with these treatments. In particular, qualitative studies have been useful for analyzing clients’ perceptions of a wide range of domains, including facilitators and barriers [19], acceptability [20], nonadherence [21], dropout [22], or negative effects [23] of IBTs. Nevertheless, the lack of integration of these primary studies has prevented their findings from being easily applied to new research or clinical practice.

In recent years, several developments within qualitative research have enhanced its quality standards. Moreover, the possibility of having tools to synthesize these studies may constitute a great leap forward. Metasynthesis makes it possible to draw conclusions from a wide range of primary studies that may focus on the same phenomenon from different perspectives using diverse qualitative methodologies in different contexts. Hence, metasynthesis can contribute to knowledge construction by revealing new insights from a detailed analysis of a broad range of findings [24]. Furthermore, these studies can identify research gaps, develop new theoretical and conceptual models, and provide evidence to further improve health interventions, in this case, psychological interventions [25].

As Timulak [26] explained, there are two kinds of metasyntheses: Those that aim to provide an assessment of the influence of a certain method on the results reported in primary studies and those that aim to increase knowledge about a particular phenomenon. This study follows the latter approach.

In addition, by drawing on the subjective and interpretative nature of qualitative research and not being merely aggregative, this type of synthesis can contribute to a more plausible understanding of reality and enhance its complexity (eg, by highlighting differences and discrepancies) [27].

### Study Aims

The goal of this metasynthesis is to review qualitative research exploring the IBT experiences of clients with mental disorders. Furthermore, this study aims to synthesize and report these experiences to foster a deeper understanding of their experiences and improve the design of the next generation of IBTs. Our study takes into consideration client preferences that constitute 1 of the 3 legs incorporated by the American Psychological Association Presidential Task Force on Evidence-Based Practice [28]. Thus, new insights can emanate from such endeavor.

## Methods

### Study Registration

This metasynthesis was registered with the International Prospective Register of Systematic Reviews, registration number: CRD42018079894). The protocol was written according to the Enhancing Transparency in Reporting the Synthesis of Qualitative Research (ENTREQ statement) [27]. Instead, of using the Population, Intervention, Control and Outcomes (PICO) criteria, we used the Sample, Phenomenon of Interest, Design, Evaluation, Research type (SPIDER) tool, which was developed specifically for the synthesis of qualitative evidence [29].

### Criteria for Study Inclusion

We will consider all primary qualitative studies examining the clients’ experiences (eg, facilitators or barriers) of an IBT for adults (18-65 years) with a mental disorder disorders. We will consider “mental disorders” to be all those documented in the principal manuals of diagnosis, such as the Diagnostic and Statistical Manual of Mental Disorders 4^th^ [30] or 5^th^ edition [31] or the International Classification of Diseases 10th edition [32]. Medical conditions other than mental disorders will be excluded. To be included, a study needs to present a qualitative analysis following established methodological criteria (eg, a descriptive and interpretative approach), and the data should be based on the reports (eg, interviews, focus groups) by participants who have undergone an IBT. In the case of mixed-methods studies, qualitative results will only be considered if they can be clearly separated from the quantitative data. Studies of completers and noncompleters will be considered, including all periods of follow-up. Additionally, eligible articles will be published in English and Spanish. There will be no restriction on the search period. As previously mentioned, all interventions delivered through web platforms or smartphone-based interventions (eg, Ecological Momentary Interventions) will be considered IBTs, regardless of the theoretical framework underlying the IBT itself.

Regarding the exclusion criteria, studies that do not follow qualitative data collection methods or do not use IBT as the intervention tool will not be included. Following Timulak’s recommendations [26] for enhancing quality control, only published data will be considered. Hence, conference abstracts, unpublished manuscripts, or thesis dissertations will not be included. Moreover, studies not published in peer-reviewed journals will also be excluded.

### Search Strategy

All articles will be identified through the following databases: PubMed, PsycINFO, and Web of Science. For each search string, the different aspects included in the SPIDER tool will be considered. In addition, back-tracking of references from relevant articles will also be searched for additional studies. We will also identify unpublished literature through Google searches with the same keywords and by contacting experts identified in the search of published literature.

Table 1 shows the SPIDER search strategy, and Multimedia Appendix 1 shows the PUBMED search strategy.

### Selection of Studies

Two reviewers will complete all database searches, and the results obtained will be entered into Mendeley folders. All duplicates will be removed. Later, one reviewer will screen all the studies to discard irrelevant studies that may have been subject to inclusion. Next, two independent reviewers will screen all the remaining titles and abstracts to identify potentially relevant articles and then review the full texts of the relevant articles to determine eligibility. A third reviewer will resolve any discrepancies.

### Quality Assessment

Two independent reviewers will assess the included studies following the Critical Appraisal Skills Programme checklist for qualitative research. This checklist allows qualitative research evidence to be appraised systematically, offering the reviewer guidance on study results, their validity, and their transferability [33]. Two independent reviewers will screen the final selection of primary studies, and a third senior reviewer (the last author of this study) will resolve any discrepancies.

### Data Extraction

All reviewers on the research team will independently extract data for each study using an Excel template to include fundamental information such as author, year, aim of the study, number of participants, gender, setting or qualitative technique used. The whole study will be considered for the analysis, although the results and discussion sections will be given priority for the data extraction since they contain the core data in each study.

**Table 1:** Sample, Phenomenon of Interest, Design, Evaluation, Research type search strategy.

Content	Description	Examples of search terms
Sample	Clients aged between18 and 65 years. Clients with diagnosis of mental disorder by the Diagnostic and Statistical Manual of Mental Disorders or International Classification of Diseases undergoing IBT^a^.	“adult” OR “client’” OR “user’” OR “patient’.” “mental disorder” OR “psychological disorder.”
Phenomenon of interest	Clients’ experiences or perceptions of a vast array of domains undergoing an IBT.	“facilitators” OR “barriers” OR “acceptability” OR “adherence” OR “dropout” OR “negative effects.” “Internet” OR “Internet based treatments” OR “online treatments” OR “Internet interventions” OR “e-therapy” OR “web based” OR “self-applied” OR “blended” OR “computer-based” OR “smartphone based intervention.”
Design	Studies that allow the extraction of qualitative data.	“Interviews” OR “focus groups” OR “questionnaire” OR “survey” OR “e-mail.”
Evaluation	Experiences, perspectives, insights, motivations or views of participants undergoing an IBT (outcome measures).	“experience” OR “view” OR “opinion” OR “attitude” OR “perception” OR “belie*” OR “belief” OR “feeling.”
Research type	Qualitative studies or mixed-methods studies.	“qualitative” OR “mixed method.”

^a^IBT: internet-based treatment.

### Data Analysis

**Figure 1:**
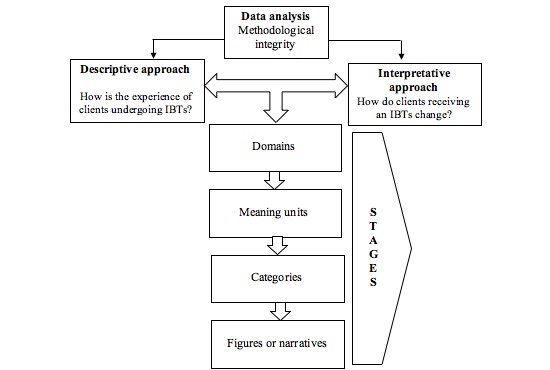
Methodological stages of the metasynthesis. IBT: internet-based treatment.

Following the guidelines of Elliott and Timulak [34], we will adopt a descriptive and interpretative approach. This method makes it possible to include a more phenomenological perspective by first describing the primary studies in detail, then complementing this description with a hermeneutical perspective that goes beyond the given information to reach new conclusions. Specifically, this approach includes four stages in which the data analysis process is carried out. First, the collected data are ordered into domains, which represent high-order conceptualizations of the phenomenon. Second, meaning units are outlined that represent the smallest understandable piece of information from the data. Domains provide a conceptual and flexible framework for the data and can be modified by the researcher until they fit the data. Third, the meaning units are organized into categories and classified in the existing domains. The creation of categories is an interpretative process in which the researcher will strive to respect the data and use category labels close to the original language provided by the participants [35]. If there are similarities between the established categories, second-order categories could be organized. Finally, the results will be presented in different forms, such as figures or narratives, to better grasp the phenomena. We will ensure methodological integrity (in terms of fidelity and utility) by following the recommendations of Levitt, Motulosky, Wertz, Morrow, and Ponterotto [36]. Thus, the different considerations at both the data collection and data analysis levels will be fulfilled. All the researchers involved in the analytic process will have extensive experience in the use of technologies to deliver psychological treatments. Figure 1 shows the data analysis process that will be conducted. In total, 5 researchers (authors 1 to 5) will be in charge of the data analysis. Each researcher will develop a domain structure that will finally be discussed in an iterative process until a consensus among all the alternatives is met. To delineate meaning units, the final number of primary studies will be divided among the coders while still ensuring that every study will be coded by two researchers in order to guarantee methodological rigor. For this second step, an iterative process will also be conducted to reach a consensus. Additionally, through this iterative process, categories that capture the fundamental sense of the meaning units will be elaborated [26].

## Results

The project was initiated in 2017 and searches were completed in 2018. Data analysis is currently under way and the first results are expected to be submitted for publication in 2019.

## Discussion

### Principal Findings

Although an abundance of qualitative studies has been published in recent years, few studies have attempted to synthesize the results. This synthesis is an essential step toward outlining more general conclusions about the experiences of clients undergoing IBTs. As long as available, IBTs do not differ much (at least in their general features), the overall experiences can be consistently integrated. In particular, qualitative studies have focused on studying topics such as facilitators, barriers, acceptability, adherence, dropouts, and negative effects. This metasynthesis will more accurately weigh the extent to which different topics related to client experiences have been addressed, in addition to establishing taxonomy within each of these topics in order to foster its use in new research studies and in the clinical application of IBTs.

Our findings will make it possible to create a hierarchy of domains based on their relevance across all the primary studies. In this regard, a conceptual framework of the barriers and facilitators perceived by the clients will be developed. Implications and recommendations for clinical practice, research, and training will be suggested.

### Potential Sources of Limitations

First and foremost, as in any other kind of review, the findings will depend on the quality of the primary studies included. Although there will be a particular emphasis on assessing the quality of the primary studies, it is a difficult task to accurately assess these kinds of studies. Moreover, there may be relevant studies in other languages that will not be included because only English and Spanish articles will be considered.

Furthermore, metasynthesis is a powerful tool for drawing overall conclusions from a certain topic. However, it may be subject to the potential underrepresentation of the richness of primary studies given that the material of analysis is not the raw data of each included study.

## Appendix

Multimedia Appendix 1 PubMed search string.

## Abbreviations

IBT: internet-based treatment
SPIDER: Sample, Phenomenon of Interest, Design, Evaluation, Research type
