# Supplementary material for: Clients’ Experiences With Internet-Based Psychological Treatments for Mental Disorders: Protocol for a Metasynthesis of Qualitative Studies
Source: JMIR Res Protoc. 2018 Nov 21;7(11):e183. doi: 10.2196/resprot.9722 (PMC6282014; doi:10.2196/resprot.9722)
Supplement: Multimedia Appendix 1 [file resprot_v7i11e183_app1.pdf]

Multimedia Appendix 1. PUBMED search strategy.

Number	Search terms
#1	psychological intervention
#2	psychological treatment
#3	#1 or #2
#4	Internet
#5	internet-based
#6	internet intervention
#7	internet treatment
#8	internet based treatment
#9	internet based intervention
#10	online treatment
#11	online intervention
#12	e therapy
#13	web based
#14	self report
#15	blended
#16	computer based
#17	smartphone
#18	mobile phone
#19	cell phone
#20	iphone
#21	android
#22	mhealth
#23	mobile application
#24	phone application
#25	#4 or #5 or #6 or #7 or #8 or #9 or #10 or #11 or #12 or #13 or #14 or #15 or #16 or #17 or #18 or #19 or #20 or #21 or #22 or #23 or #24
#26	qualitative study
#27	qualitative research
#28	focus group
#29	mixed method
#30	interview*
#31	questionnaire*
#32	survey
#33	#26 or #27 or #28 or #29 or #30 or #31 or #32
#34	facilitator*
#35	barriers
#36	complain*
#37	noncomplain*
#38	adher*
#39	nonadher*
#40	dropout*
#41	dropout
#42	acceptability
#43	negative effects
#44	#34 or #35 or #36 or #37 or #38 or #39 or #40 or #41 or #42 or #43
#45	experience
#46	view*

#47	narrative*
#48	opinión*
#49	perspective*
#50	belie*
#51	belief
#52	feeling
#53	#45 or #46 or #47 or #48 or #49 or #50 or #51 or #52
#54	mental disorder*
#55	psychological disorder*
#56	anxiety disorder
#57	depressive disorder
#58	depression
#59	anxiety
#60	schizophrenia
#61	substance use disorder
#62	addictive disorder
#63	bipolar disorder
#64	obsessive-compulsive disorder
#65	posttraumatic stress disorder
#66	eating disorder
#67	sleep disorder
#68	disruptive disorder
#69	impulse-control disorder
#70	conduct disorder
#71	sexual disorder
#72	#47 or #48 or #49 or #50 or #51 or #52 or #53 or #54 or #55 or #56 or #57 or #58 or #59 or #60 or #61 or #62 or #63 or #64
#73	#3 and #25 and #33 and #44 and #53 and #72
